# Response of Archaeal Communities in the Rhizosphere of Maize and Soybean to Elevated Atmospheric CO_2_ Concentrations

**DOI:** 10.1371/journal.pone.0015897

**Published:** 2010-12-29

**Authors:** David M. Nelson, Isaac K. O. Cann, Roderick I. Mackie

**Affiliations:** 1 Appalachian Laboratory, University of Maryland Center for Environmental Science, Frostburg, Maryland, United States of America; 2 Institute for Genomic Biology, University of Illinois, Urbana, Illinois, United States of America; 3 Department of Animal Sciences, University of Illinois, Urbana, Illinois, United States of America; 4 Department of Microbiology, University of Illinois, Urbana, Illinois, United States of America; Argonne National Laboratory, United States of America

## Abstract

**Background:**

Archaea are important to the carbon and nitrogen cycles, but it remains uncertain how rising atmospheric carbon dioxide concentrations ([CO_2_]) will influence the structure and function of soil archaeal communities.

**Methodology/Principal Findings:**

We measured abundances of archaeal and bacterial 16S rRNA and *amo*A genes, phylogenies of archaeal 16S rRNA and *amo*A genes, concentrations of KCl-extractable soil ammonium and nitrite, and potential ammonia oxidation rates in rhizosphere soil samples from maize and soybean exposed to ambient (∼385 ppm) and elevated (550 ppm) [CO_2_] in a replicated and field-based study. There was no influence of elevated [CO_2_] on copy numbers of archaeal or bacterial 16S rRNA or *amo*A genes, archaeal community composition, KCl-extractable soil ammonium or nitrite, or potential ammonia oxidation rates for samples from maize, a model C_4_ plant. Phylogenetic evidence indicated decreased relative abundance of crenarchaeal sequences in the rhizosphere of soybean, a model leguminous-C_3_ plant, at elevated [CO_2_], whereas quantitative PCR data indicated no changes in the absolute abundance of archaea. There were no changes in potential ammonia oxidation rates at elevated [CO_2_] for soybean. Ammonia oxidation rates were lower in the rhizosphere of maize than soybean, likely because of lower soil pH and/or abundance of archaea. KCl-extractable ammonium and nitrite concentrations were lower at elevated than ambient [CO_2_] for soybean.

**Conclusion:**

Plant-driven shifts in soil biogeochemical processes in response to elevated [CO_2_] affected archaeal community composition, but not copy numbers of archaeal genes, in the rhizosphere of soybean. The lack of a treatment effect for maize is consistent with the fact that the photosynthesis and productivity of maize are not stimulated by elevated [CO_2_] in the absence of drought.

## Introduction

Microorganisms play a critical role in the cycling of carbon (C) and nitrogen (N) in soils, which may be affected by environmental changes, such as the rapidly rising concentration of carbon dioxide in the earth's atmosphere ([CO_2_]) [1]. Most previous studies concerning the effects of elevated [CO_2_] on soil microbial community composition have focused on bacteria and/or fungi. These studies have found significant [2], [3], [4], as well as insignificant [5], [6], [7], [8], effects of elevated [CO_2_]. However, there is a growing appreciation for the importance of archaea to the global C and N cycles [9], [10]. Besides the known involvement of euryarchaeota in methane production [11], recent studies have revealed the ability of crenarchaeota to oxidize ammonia to nitrite [12], [13], [14], a process originally thought to be performed only by bacteria [9]. Studies have found crenarchaeal 16S rRNA and *amo*A (the A subunit of ammonia monooxygenase) gene sequences in a variety of settings, including natural and agricultural soils [15], [16], [17], suggesting their widespread distribution and previously unrecognized contributions to soil biogeochemical cycles.

Free air CO_2_ enrichment (FACE) sites enable assessment of the effects of elevated [CO_2_] on ecosystem processes in field settings, without the disruption of the natural soil-plant-atmosphere continuum that occurs with environmental or open-top chambers [18]. Such FACE studies reveal important differences in the responses of the two major plant photosynthetic types (C_3_ and C_4_) to elevated [CO_2_]. Results from a FACE facility in central Illinois, USA, indicate that the photosynthesis and productivity of maize, a C_4_ plant, are not directly stimulated by elevated [CO_2_] (550 ppm) unless drought stress occurs [19]. In contrast, photosynthesis is directly stimulated by elevated [CO_2_] in soybean, a C_3_ plant [20]. When soybean is grown at elevated [CO_2_] increases in photosynthetic carbon uptake of ∼20% [21], aboveground net primary production of ∼18%, and seed yield of ∼15% have been observed [22].

In addition to aboveground changes, FACE studies indicate that elevated [CO_2_] affects belowground processes and soil N dynamics. Root biomass of soybean has been shown to increase ∼30% in response to elevated [CO_2_] at the central Illinois FACE site [23], whereas extractable amino N and ammonium concentrations in soils have been shown to decline [24]. It is also thought that plants hosting N_2_-fixing bacteria, such as soybean, may allocate additional C to the symbiosis in exchange for additional N [25], [26]. Belowground changes in response to elevated [CO_2_] have the potential to alter the structure and function of soil archaeal communities. For example, a recent study of soils beneath trembling aspen (*Populus tremuloides*), a C_3_ tree, at a FACE site in northern Wisconsin, USA, found a significant decline in the copy numbers of archaeal 16S rRNA genes at elevated, relative to ambient, [CO_2_] [27]. However, whether the response of soil archaeal communities associated with the roots of aspen to elevated [CO_2_] also occur with shorter-lived, non-woody, and annually-rotated plants, including maize [28] and soybean [29], is unknown.

The objective of this study was to evaluate the influence of ambient vs. elevated [CO_2_] on rhizosphere soil archaea, potential ammonia oxidation rates, and extractable ammonium and nitrite concentrations in a maize-soybean agroecosystem. We hypothesized that because maize uses the C_4_ photosynthetic pathway, there would be no change in abundances of archaeal and bacterial 16S rRNA and *amo*A gene sequences or phylogenies of archaeal 16S rRNA and *amo*A gene sequences between maize grown at ambient and elevated [CO_2_]. We further hypothesized that there would be no changes in potential ammonia oxidation rates or extractable ammonium and nitrite concentrations between maize grown at ambient and elevated [CO_2_]. In contrast, we hypothesized that because soybean uses the C_3_ photosynthetic pathway and has a symbiotic relationship with N_2_-fixing bacteria, elevated [CO_2_] would lead to shifts in the above variables through changes in factors such as plant inputs to soils. Maize-soybean agroecosystems dominate the landscape of the midwestern US, and they provide a model system for investigating the influence of plant-microbe interactions on C and N cycling under global change scenarios.

## Materials and Methods

### Field Site, Cultivation, and FACE System

Samples for this study were collected from the SoyFACE (Soybean Free Air Concentration Enrichment) facility in Champaign, IL, a 32-ha field of maize (*Zea mays* L. cv 34B43 [Pioneer Hi-Bred International, Des Moines, IA]) and soybean (*Glycine max* L. Merr. cv. 93B15 [Pioneer Hi-Bred International]). SoyFACE was established in 2001, growing maize and soybean in annual rotation following local agricultural practices. The soils are from the Drummer-Flanagan series and classified as fine-silty, mixed, mesic Typic Endoaquolls [30]. Spring tillage is performed before planting of both crops; only maize crop residue is tilled in the fall. Fields to be planted with maize received a treatment of 28% liquid urea at a rate of 68 kg/acre prior to planting, whereas soybean fields received no supplemental N.

The procedures for operation and crop cultivation have been previously reported in detail [31]. After sowing, the infrastructure for the CO_2_ treatment was installed in a randomized complete block design (n = 4). Each block contained two 20-m-diameter plots. One plot received an ambient [CO_2_] treatment and a second plot received an elevated [CO_2_] treatment of 550 ppm, the anticipated [CO_2_] for the year 2050 [32]. The infrastructure consists of rings with horizontal pipes (with holes along the pipes) that release CO_2_ on the upwind side of the plot. CO_2_ release is controlled by measured [CO_2_] in the plots, wind direction, and wind speed. The ambient plots are surrounded by the same infrastructure, but do not receive the CO_2_ treatment. Thus we utilized a total of 16 plots (i.e. 4 maize/ambient [CO_2_], 4 maize/elevated [CO_2_], 4 soybean/ambient [CO_2_], and 4 soybean/elevated [CO_2_]). [CO_2_] in the elevated plots is within ±20% of the target for 90% of the time [31].

### Sample Collection and DNA Extraction

We collected rhizosphere soil samples from the roots of two plants from each plot (32 total samples) over the course of two days during the peak of the growing season in mid-August 2006 and late-July 2008. All samples from each crop were collected on a single day each year, and thus they do not capture potential temporal (e.g. within season) fluctuations in the variables we measured. Each plant was gently uprooted, shaken, and soil adhering to the roots was collected. We did not sample in 2007 because the infrastructure for treating maize with [CO_2_] is only installed biennially.

Within 24 hours of collection portions of the soil samples to be utilized for molecular analyses were freeze dried, sieved to remove roots and nodules, ground, and stored at −80°C before DNA extraction. DNA was extracted from 0.25 g of soil from each plant root using a PowerSoil™ DNA Isolation Kit (MoBio Labs, Inc., Carlsbad, CA). DNA concentrations were determined using the Quant-iT PicoGreen dsDNA kit (Invitrogen, Carlsbad, CA). The two DNA samples from each plot were combined in equimolar amounts, yielding 16 total DNA samples per sampling year for molecular analysis.

### Real-Time Quantitative PCR

The extracted DNA was subject to real-time quantitative PCR (q-PCR) [33], targeting fragments of the 16S rRNA and *amo*A genes using primers specific to archaea and bacteria for each gene. Archaeal and bacterial 16S rRNA gene sequences were amplified using primers A109f [34], [35] and PARCH519r [36], and 341F and 534R [37], respectively. Cycling conditions were as follows: 2 min at 50°C, 10 min at 95°C, 50 cycles of 16 s at 95°C and 30 s at x°C, followed by a plate read, where x = 59 for archaeal 16S rDNA and x = 60 for bacterial 16S rDNA. Portions of archaeal and bacterial *amo*A gene sequences were amplified using primers *amo*196F and *amo*277R and *amo*A-1F and *amo*A-2R, respectively [16]. Cycling conditions were as above, except that annealing steps of 40 s at 53°C and 30 s at 54°C were used, respectively. All reactions were performed in 10 ul volume with 1 ng of template DNA using SYBR Green as a fluorescent dye on a 384 well Applied Biosystems 7900HT Fast Real-Time PCR System (Applied Biosystems Inc., Foster City, CA).

Standards of the respective genes for qPCR were obtained by amplifying DNA from soil samples using the above primers with the Roche FastStart High Fidelity PCR System. The amplification products were visualized on 1% agarose gels in 1× TAE buffer and purified from the gel using a QIAquick PCR purification kit (QIAGEN Sciences, Valencia, CA). The purified PCR products were cloned into pGEM-T Easy (Invitrogen). Single colonies, verified for the expected insert using PCR, and sequenced to confirm the identity of the expected gene, were grown in 1 ml of LB medium supplemented with ampicillin (100 µg/ml) overnight. Subsequently, the plasmids were extracted using a QIAprep Spin miniprep kit (QIAGEN Sciences). The concentrations of the purified plasmids were determined using the Quant-iT PicoGreen dsDNA kit. Ten-fold dilution series of the plasmids were used to generate standard curves with ranges of approximately 10^9^ to 10^1^ copies µl^−1^. Sample copy numbers were determined by relating cycle threshold (Ct) values to known copy numbers in the standards. All reactions were repeated to confirm results. The *R^2^* values of all standard curves were >0.97.

### Creation of Amplicon Libraries and Sequencing

DNA from the 2006 samples was used for amplicon-based pyrosequencing of the archaeal 16S rRNA and *amo*A genes. Amplification of the V3 region of the archaeal 16S rRNA gene used primers A109f and PARCH519r, and amplification of a portion of the archaeal *amo*A gene used primers Arch-*amo*F2 and Arch-*amo*AR [38], [39]. Primer Arch-*amo*F2 (5′- GGTNGCVAARRGHGCWTGG -3′) was designed to amplify inside of the region targeted by primer Arch-*amo*F [38] and Arch-*amo*AR in order to broaden the diversity of the *amo*A genes amplified and to decrease the size of the amplicons to <500 bp, as recommended for sequencing using Roche's 454 GS-FLX system (454 Life Sciences, Branford, CT). To the 5′ end of both forward primers we added Roche's fusion primer A (5′-GCCTCCCTCGCGCCATCAG-3′), and to the reverse primers we added Roche's fusion primer B (5′-GCCTTGCCAGCCCGCTCAG-3′) followed by one of four unique ten-base barcodes recommended by Roche (5′-ACGAGTGCGT-3′, 5′-ACGCTCGACA-3′, 5′-AGACGCACTC-3′, or 5′-AGCACTGTAG-3′).

The Roche FastStart High Fidelity PCR System was used for amplification following the manufacturer's instructions with the following thermocycler procotol: 2 min at 95°C; 25 cycles consisting of 30 s at 94°C, 1 min at x°C, and 1 min at 72°C; and 7 min at 72°C, where x = 59 for the archaeal 16S-V3 rRNA gene and x = 54 for the archaeal *amo*A gene. Twelve independent PCRs (of 30 µl each) were performed for each of the 16 samples to amplify the archaeal 16S rRNA gene and twenty-four independent PCRs (of 30 µl each) were performed for each of the 16 samples to amplify the archaeal *amo*A gene. For each sample the independent PCRs were combined and purified using a QIAquick PCR Purification Kit. The DNA was run on a 1% TAE gel and the correct-sized products were excised, purified, and eluted in 1× TE. The DNA concentration in each sample was quantified using the Quant-iT PicoGreen dsDNA kit, and DNA quality was assessed using a Bioanalyzer with a DNA1000 chip.

A LR25 sequencing plate was divided into four regions. To each region a master DNA pool, created by combining eight purified PCR products (four samples from the 16S rRNA gene and four samples from the *amo*A gene) in equimolar ratios, was added. Pyrosequencing proceeded from fusion primer B at the University of Illinois Biotechnology Center using Roche's 454 GS-FLX system. Sequences were assigned to their original samples using the gene-specific and barcode sequences. Primer and barcode sequences were trimmed and low-quality sequences were removed from the dataset using established criteria for evaluating the quality of pyrosequencing reads [40], [41], including removal of sequences with Q25 scores <25, incorrect tag or primer sequences, non IUPAC characters, or read lengths < or > than the 2σ range of all reads for each gene. These quality-control procedures eliminated an average of 11% of all 16S rDNA sequences and 6% of all *amo*A sequences.

### Analysis of 16S rDNA Sequence Data

The 16S rRNA gene sequences from each sample were analyzed with the RDP classifier [42], and sequences identified as being bacterial in origin were removed from the dataset (Table S1). Sequences from each sample were aligned using Infernal [43] with the RDP's pyrosequencing pipeline [44] and the NAST alignment tool [45]. The RDP and NAST multiple sequence alignments for each sample were merged (http://acai.igb.uiuc.edu/bio/merge-nast-infernal.html), and manually edited in Jalview [46]. The sequences were then clustered (max. distance = 3%, step size = 3) using the complete-linkage clustering method in RDP, which uses a furthest neighbor clustering approach. The alignment and cluster files were used for dereplication, rarefaction, and Chao1 analyses (max. distance = 3) in RDP. The relative abundances of the representative sequences from the dereplication analyses were recorded. The multiple sequence alignments containing representative sequences from all 16 samples were combined into a single multiple alignment, which was used to create 100 bootstrapped maximum likelihood (ML) trees using RAxML version 7.0.4 [47] with the GTRGAMMA nucleotide substitution model. The ML trees were rooted using bacterial 16S rRNA gene sequences from *E*. *coli* J01859 and *Nitrosomonas europaea* AL954747. The trees were visualized using iTOL [48].

We used the best-scoring ML tree for downstream multivariate analysis, because unlike taxonomy-based methods (e.g. RDP's classifier) that assign names to sequences, the ML tree enables evolutionary relationships to be considered in comparisons among samples. The best-scoring ML tree was analyzed using weighted and unweighted principle coordinates analysis (PCA) in Unifrac [49] to determine if statistical differences exist among our treatments. Weighted PCA accounts for the relative abundance of a particular sequence in a sample.

### Analysis of amoA Sequence Data

The *amo*A nucleotide sequences from each sample were subject to dereplication, rarefaction, and Chao1 analyses using FastGroup II with a percent sequence identity (with gaps) of 97% [50]. The representative sequences from each sample were then dereplicated for all maize and soybean plots. The relative abundances of the representative sequences were recorded at each step. The second sets of representative sequences were aligned using CLUSTAL W [51] in the MEGA 4.0 software package [52] and then manually edited in Jalview. FastTree 2.1.2 was used to infer an approximately-ML phylogenetic tree [53], which was rooted using a bacterial *amo*A gene nucleotide sequence. The tree was visualized and analyzed as described above for the 16S rDNA data. We analyzed nucleotide sequences, rather than predicted amino acid sequences, to focus on genetic heterogeneity among samples.

### Biogeochemical and Soil Moisture Measurements

Ammonium and nitrite were extracted, and potential nitrification assays performed within 24 hours of sampling using a fresh portion of the 2008 samples from which roots and nodules were removed. Ammonium and nitrite were extracted from 1 g soil using 2 M KCl, and their concentrations were measured using the phenolhypochlorite and sulfanilamide spectrophometric methods, respectively [54], and used as a measure of N in soil solution that is available to plants and microbes. Potential ammonia oxidation assays were performed using the chlorate inhibition method, as described in He *et al.* [39]. Briefly, 1 g of soil was added to a 50 ml centrifuge tube containing 20 ml phosphate buffer solution and 1 mM ammonium sulfate. Potassium chlorate (10 mM final concentration) was added to inhibit nitrite oxidation. The suspension was incubated in the dark at room temperature for 24 hours and nitrite was extracted with 2 M KCL [39] and analyzed as above. In addition, potential ammonia oxidation assays were performed as above except with antibiotics (100 ml/L ampicillin and streptomycin) added to the assays to reduce potential ammonia and nitrite oxidation by bacteria. Soil pH was measured following extraction with 0.01 M CaCl_2_ [55].

Volumetric soil water content was measured within 24 hours of rhizosphere sampling using a capacitance probe (Diviner-2000, Sentek Sensor Technologies, Australia). Within each replicate plot, measurements were taken at location between the crop rows and two locations within the crop rows. Data are reported as the average soil water content between 5–25 cm and 25–55 cm depth, based on data collection at depth increments of 10 cm.

### Statistical Analyses and Nucleotide Sequence Accession Numbers

The qPCR, PCA, ammonium, nitrite, and potential ammonia oxidation rate data were analyzed using one-way ANOVAs in PAST [56] to test for significant differences among plant/[CO_2_] combinations. For each crop, growing season, and soil layer, the soil moisture data were analyzed using a complete block analysis of covariance with day of year as the repeated measure, [CO_2_] treatment as a main effect, block as a random factor and early season saturated soil H_2_O in each plot at the beginning of the growing season as the covariate (Mixed Procedure, SAS 9.1, The SAS Institute, Raleigh, NC, USA). Treatment effects on the specific measurement dates were assessed with pair-wise comparisons using the pdiff option in the Mixed Procedure. Sequences obtained in this study have been deposited in the EMBL Short Read Archive (accession #ERP000203).

## Results

**Figure 1:**
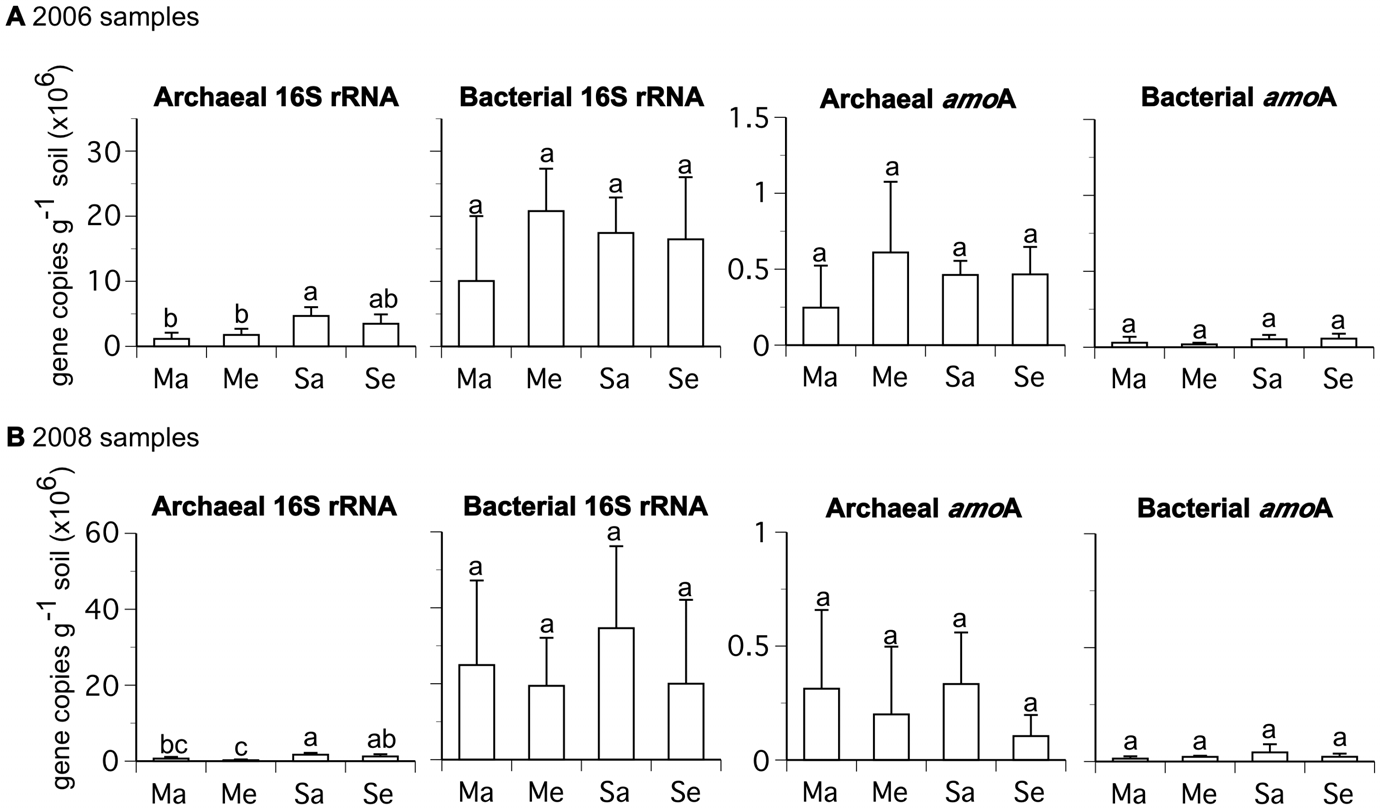
Abundance of archaeal and bacterial 16S rRNA and *amo*A genes from rhizosphere soil samples collected during the a) 2006 and b) 2008 growing seasons based upon quantitative PCR. The abbreviations are as follows: maize ambient [CO_2_] (Ma), maize elevated [CO_2_] (Me), soybean ambient [CO_2_] (Sa), and soybean elevated [CO_2_] (Se). Mean values (+/− one standard deviation) are shown. Letters indicate statistical differences among treatments.

Mean copy numbers among samples for the archaeal 16S rRNA, bacterial 16S rRNA, archaeal *amo*A, and bacterial *amo*A gene sequences, ranged from 0.2–4.6×10^6^, 10.1–34.6×10^6^, 1.1–6.1×10^5^, and 1.3–5.7×10^4^ per gram of dry soil, respectively. No statistical differences existed in the copy numbers of the bacterial 16S rRNA, archaeal *amo*A, or bacterial *amo*A gene sequences among treatments for samples from either year. However, the archaeal 16S rRNA gene was significantly more abundant in soybean at ambient [CO_2_] than maize at ambient [CO_2_] in both years. There was no difference in the abundance of the archaeal 16S rRNA gene between maize at ambient and elevated [CO_2_] or soybean at ambient and elevated [CO_2_] (Fig. 1).

**Figure 2:**
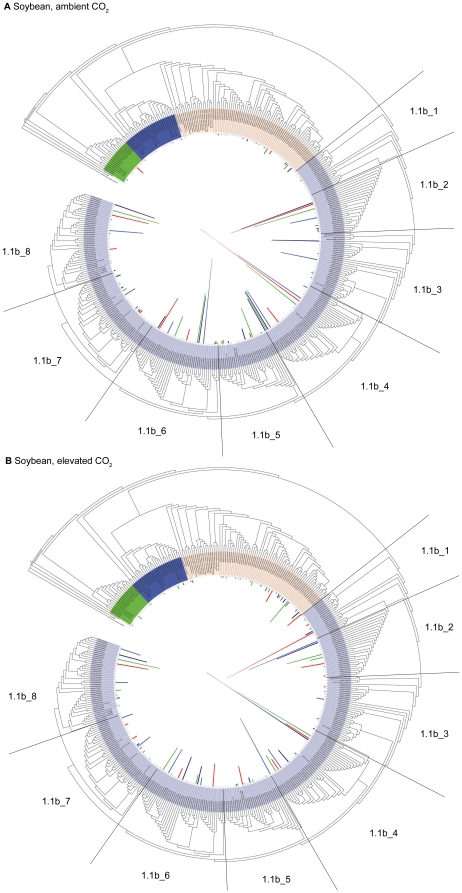
Phylogenetic trees of archaeal 16S rRNA gene sequences from rhizosphere soil samples from soybean grown at ambient and elevated [CO_2_]. The colored bars represent the relative abundances of representative sequences from individual plots for each [CO_2_] treatment (Table S1). Each colored bar in each phylogenetic tree represents data from a different plot. Starting from the root, the colors for the leaf ranges indicate crenarchaeota group 1.1a (green), crenarchaeota group 1.1c (dark blue), euryarchaeota (tan), and crenarchaeota group 1.1b (light blue). Clusters within crenarchaeota group 1.1b were further divided into arbitrarily named groups as shown (e.g. 1.1b_1). Only branches with bootstrap support >60 are shown, and identical branch lengths are shown.

**Figure 3 pone-0015897-g003:**
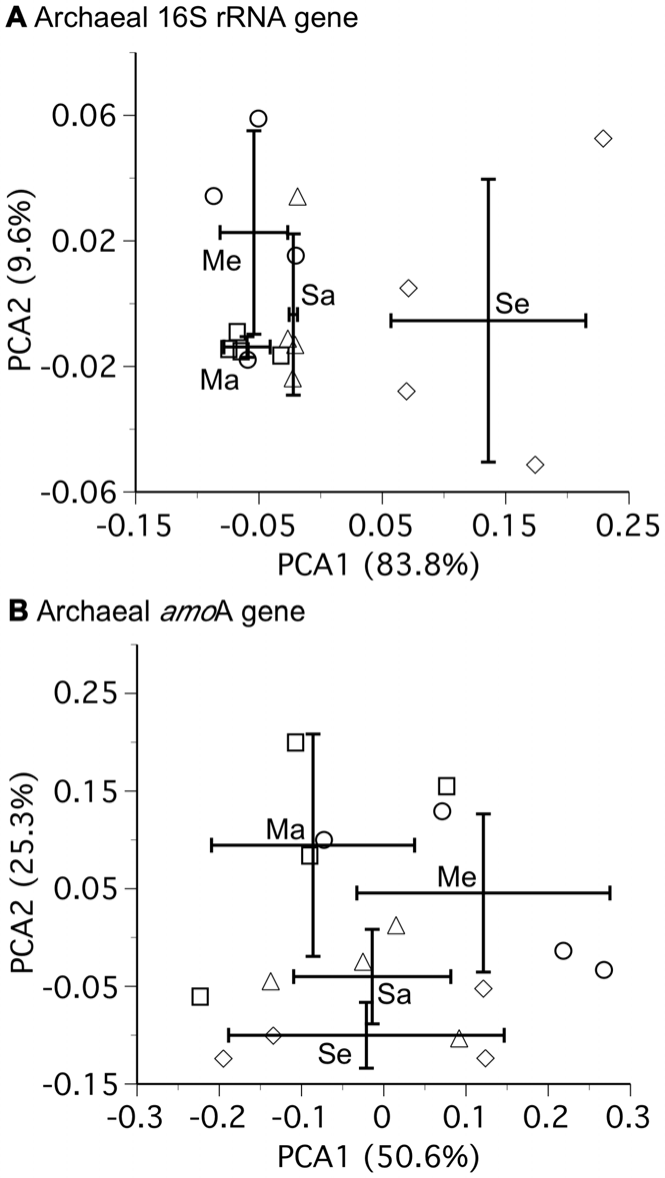
Principle component analysis of a) archaeal 16S rRNA and b) archaeal *amo*A gene sequence data from phylogenetic trees (Figs. 2, S3, S5) of samples collected during the 2006 growing season. PCA was performed in UniFrac [49] using abundance weights. The symbols are as follows: soybean elevated [CO_2_] (Se), diamonds; soybean ambient [CO_2_] (Sa), triangles; maize elevated [CO_2_] (Me) circles; maize ambient [CO_2_] (Ma), squares. The mean PCA scores (+/− one standard deviation) are shown for each axis. For a), no plant/[CO_2_] combinations differ on PCA2, whereas Se and Sa are statistically different (p<0.05) from each other and Me and Ma on PCA1. There is no difference (p = 0.72) between Me and Ma on PCA1. For b), no plant/[CO_2_] combinations differ on PCA1, whereas Se is significantly different from Ma (p<0.05) on PCA2. Se is marginally different from Me (p = 0.08), and Sa from Ma (p = 0.11), on PCA2.

**Figure 4 pone-0015897-g004:**
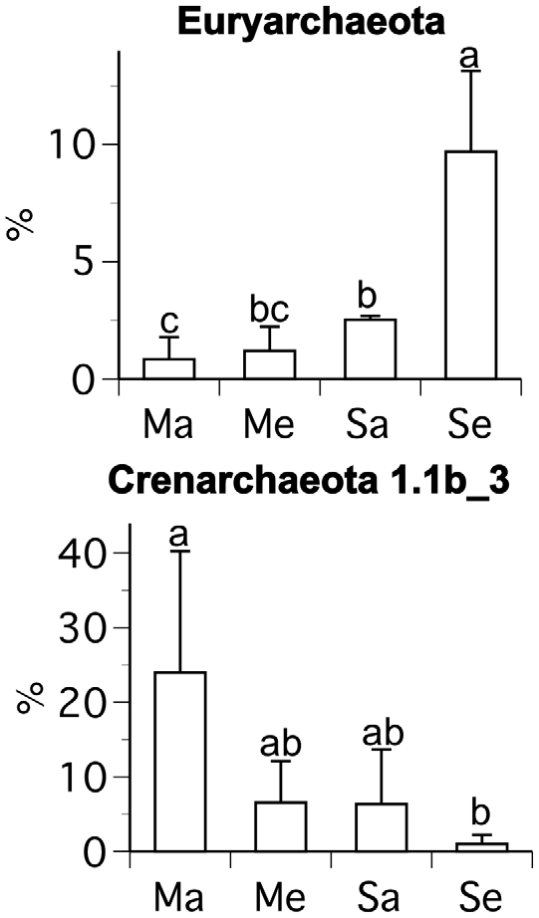
Percentages of archaeal lineages (identified in Figs. 2, S3) for which there were statistical differences among plant/[CO_2_] combinations. The abbreviations are as follows: maize ambient [CO_2_] (Ma), maize elevated [CO_2_] (Me), soybean ambient [CO_2_] (Sa), and soybean elevated [CO_2_] (Se). Mean values (+/− one standard deviation) are shown. Letters indicate statistical differences among treatments.

The RDP classifier indicated that >99.7% of the recovered 16S rRNA gene sequences were archaeal in origin, which is consistent with the fact that we used primers specific to the archaeal 16S rRNA gene. The average number of reads obtained per sample was 656 (range: 270–1274) and the average read length was 252±61 bp. Chao1 richness estimates indicate an average of 15–51 archaeal operational taxonomic units in the samples based on 16S rDNA phylogenetic diversity (Table S1). Rarefaction curves did not plateau, indicating that additional diversity of archaea exists in these samples than was captured by the present sequencing effort (Fig. S1). All samples were dominated by a relatively small number of abundant taxa and larger numbers of relatively rare taxa (Figs. 2, S2). RDP's classifier and phylogenetic analysis indicated that, on average, ∼96% of the sequences were from representatives of the crenarchaeota, and particularly crenarchaeota group 1.1b (Figs. 2, S3). Unweighted PCA, based upon phylogenetic analysis of the 16S rDNA data, indicated no difference in archaeal community composition among treatments (data not shown), and weighted PCA of the 16S rDNA data indicated no difference in archaeal community composition between maize at ambient and elevated [CO_2_]. However, weighted PCA of the 16S rDNA data indicated significant differences in archaeal community composition between soybean and maize at ambient and elevated [CO_2_] and soybean at ambient and elevated [CO_2_]. The difference in community composition between soybean at ambient and elevated [CO_2_] was greater than the difference between maize and soybean at ambient [CO_2_] (Fig. 3a). These differences in community composition were partly caused by greater relative abundance of euryarchaeota (and specifically members of the class Methanomicrobia) for soybean at elevated [CO_2_] (Figs. 2–4). For example, euryarchaeota comprised an average of 9.6% of sequences from soybean at elevated [CO_2_], but only an average of 1.5% of sequences from the other plant/[CO_2_] combinations (Fig. 4). The only statistical difference in the relative abundance of sequences from clusters of crenarchaeota among treatments was lower abundance of crenarchaeota group 1.1b_3 (the names of the suffices, i.e. “_3,” used here and throughout this paper, are arbitrarily assigned) for soybean at elevated [CO_2_] than maize at ambient [CO_2_] (Figs. 2, 4, S3, S4).

The average number of reads of the archaeal *amo*A gene obtained per sample was 1378 (range: 83–2380) and the average read length was 221±38 bp. Rarefaction curves suggest greater diversity of the archaeal *amo*A gene than was captured by our sequencing effort (Fig. S1). All samples were dominated by a relatively small number of abundant *amo*A gene sequences and larger numbers of relatively rare *amo*A gene sequences (Figs. S2, S5). Unweighted PCA of the *amo*A data indicated no differences among treatments (data not shown), whereas weighted PCA indicated marginal differences between maize and soybean at ambient, and at elevated, [CO_2_]. Phylogenetic differences among our samples were overall less pronounced for the archaeal *amo*A than 16S rRNA gene (Fig. 3).

**Figure 5:**
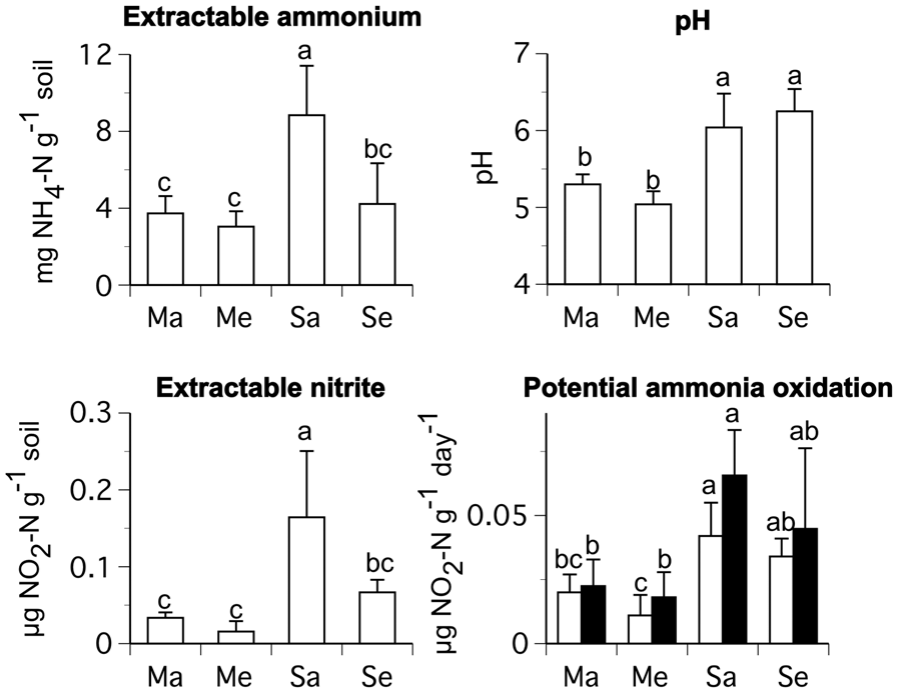
Average KCl-extractable ammonium and nitrite concentrations, pH, and potential rates of ammonia oxidation from rhizosphere soil samples collected during the 2008 growing season. The abbreviations are as follows: maize ambient [CO_2_] (Ma), maize elevated [CO_2_] (Me), soybean ambient [CO_2_] (Sa), and soybean elevated [CO_2_] (Se). Mean values (+/− one standard deviation) are shown. Letters indicate statistical differences among treatments. For potential ammonia oxidation data, closed bars indicate assays to which antibiotics were added and open bars indicate assays to which antibiotics were not added, as described in the text. Statistical comparison of potential ammonia oxidation data is among assays with antibiotics and among assays without antibiotics.

For the 2008 samples, mean KCl-extractable ammonium concentrations were 3.7–8.8 mg NH_4_-N/g soil, nitrite concentrations 0.02–0.16 µg NO_2_-N/g soil, potential ammonia oxidation rates 0.01–0.07 µg NO_2_-N/g soil/day, and pH values 5.3–6.3. Ammonium concentrations, nitrite concentrations, potential ammonia oxidation rates (with and without antibiotics added to the assay), and pH values of rhizosphere soil were significantly higher for soybean than maize at ambient [CO_2_]. Extractable ammonium and nitrite concentrations were significantly lower for soybean at elevated than ambient [CO_2_]. There was no difference in pH, ammonium concentrations, nitrite concentrations, or potential ammonia oxidation rates between maize at ambient and elevated [CO_2_]. There were also no differences in soil pH or potential ammonia oxidation rates between soybean at ambient and elevated [CO_2_] (Fig. 5). Elevated [CO_2_] did not affect soil moisture for either crop when the samples for this study were collected (Table 1) or for at least 20 days prior to our sampling dates (A. Leakey, personal communication).

**Table 1:** Soil water content (% vol/vol) of plots of soybean and maize grown at ambient and elevated [CO_2_] on dates of rhizosphere sampling during the 2006 and 2008 growing seasons.

			5–25 cm		25–55 cm	
Crop	Year	DOY	Ambient [CO_2_]	Elevated [CO_2_]	Ambient [CO_2_]	Elevated [CO_2_]
soybean	2006	227	38.9±1.3	39.5±1.3	42.8±0.4	42.2±0.4
	2008	213	30.3±0.9	31.5±0.9	39.2±1.1	38.6±1.1
maize	2006	227	32.2±1.9	34.7±1.9	38.5±0.8	40.0±0.8
	2008	198	33.4±1.3	34.4±1.3	42.0±1.0	41.5±1.0

There were no statistically significant effects of elevated [CO_2_] during these sampling periods.

## Discussion

Our results indicate that the effect of elevated [CO_2_] on rhizosphere soil archaea and KCl-extractable ammonium and nitrite concentrations varied between representatives of the two major plant photosynthetic types, maize and soybean. Consistent with our hypothesis, there was no effect of elevated [CO_2_] on maize for any of the variables measured in this study (Figs. 1, 3–5). Maize uses the C_4_ photosynthetic pathway, which is saturated at ambient [CO_2_], and thus elevated [CO_2_] does not directly stimulate photosynthesis or productivity in maize. Elevated [CO_2_] may indirectly influence C_4_ plants by increasing soil moisture during drought [57], but there was no difference in soil moisture between ambient and elevated [CO_2_] for either crop during collection of our samples (Table 1).

A possible explanation for greater relative abundance of euryarchaeota than crenarchaeota in the rhizosphere of soybean and maize at ambient [CO_2_] (Figs. 2–4) is that N_2_ -fixing bacteria live in the root nodules of soybean, whereas they do not live in maize roots. H_2_ is a by-product of N_2_ fixation by legumes [58], [59], and H_2_ is also used as a reducing agent to convert CO_2_ to methane during anaerobic respiration by methanogens. Nearly half of cultivated euryarchaeota, including those of the class Methanomicrobia, are methanogens. Thus euryarchaeota may increase in relative abundance in environments with greater H_2_ production because they are capable of metabolizing H_2_, in contrast to other archaea. Prior research also suggests that elevated [CO_2_] may lead to greater rates of N_2_ fixation and H_2_ production by some legumes [25], [60]. Thus the greater relative abundance of euryarchaeota in the rhizosphere of soybean at elevated than ambient [CO_2_] may be caused by increased H_2_ production at elevated [CO_2_]. However, rates of N_2_ fixation and H_2_ production for soybean grown at elevated versus ambient [CO_2_] at this FACE site have not been reported [25], so this hypothesis requires further investigation.

Archaeal community composition may also be influenced by pH, plant type, and/or N concentration, as suggested by prior studies [15], [39]. Soil pH was consistently higher for soybean than maize in samples from 2008, whereas concentrations of KCl-extractable ammonium and nitrite were similar between maize and soybean at elevated [CO_2_], but higher in soybean at ambient than elevated [CO_2_] (Fig. 5). Decreased extractable N concentrations at elevated [CO_2_] (Fig. 5 and [24]) may indicate increased plant uptake and/or microbial immobilization of N, as well as a possible interacting influence of pH (or plant type) and N on archaeal community composition. However, direct evaluation of such potential influences on archaeal community composition is not possible using the data reported here because our soil pH and KCl-extractable nitrogen data come from the 2008 samples, whereas the community composition data come from the 2006 samples.

Phylogenetic changes in archaeal community composition at elevated [CO_2_] in the rhizosphere of soybean could have implications for climate change and/or biogeochemical processes if different groups of archaea have varying influences on rates of soil processes, as has been demonstrated for bacteria [61], [62]. For example, a shift toward more Methanomicrobia in the rhizosphere of soybean in response to elevated [CO_2_] could enhance atmospheric warming. Previous research showed that rice paddy soils exposed to elevated [CO_2_] displayed increased methane emissions [63], [64], although soils from beneath a grass at a FACE experiment in Switzerland displayed decreased methane emissions in response to elevated [CO_2_] [65]. Recent studies implicate crenarchaea with ammonia oxidation [12], [14] and show that crenarchaeal abundance may influence rates of ammonia oxidation [39]. However, despite decreased relative abundance of crenarchaeota in the rhizosphere of soybean at elevated [CO_2_] (Fig. 4), we observed no influence of elevated [CO_2_] on potential ammonia oxidation rates (Fig. 5). Nevertheless, in agreement with our data (Figs. 2, S3), previous studies demonstrate that members of crenarchaeota group 1.1b are the dominant archaeal lineages in most soils [9], [15], [66], [67]. Thus greater knowledge of the ecology and physiology of different groups of soil crenarchaeota, and particularly group 1.1b, is required to project the effects of changes in crenarchaeal community composition on ecosystem processes. A potential target for future study are members of crenarchaeota group 1.1b_3, which inhabit rhizosphere soils [68] and have been selectively enriched in mixed culture [69].

Our finding of decreased relative abundance of crenarchaea in the rhizosphere of soybean at elevated [CO_2_] is qualitatively consistent with interpretations based upon results from soils beneath trembling aspen [27]. However, the finding from trembling aspen was driven by decreased archaeal 16S rRNA gene copy numbers at elevated [CO_2_], which is not indicated by our q-PCR data (Fig. 1). Instead, our phylogenetic data indicate a shift in archaeal community composition towards lower relative abundance of crenarchaea at elevated [CO_2_]. Together these results from both long-lived and woody (i.e. aspen), as well as annually-planted and herbaceous (i.e. soybean), C_3_ plants, indicate distinct changes in the absolute and relative abundances of rhizosphere archaea in response to plant-driven changes in soil biogeochemical processes at elevated [CO_2_]. Improving projections of changes to C and N cycling in soils in response to rising [CO_2_] will require knowledge of the functional significance of such shifts on intra- and inter-annual time-scales.

## Supporting Information

Figure S1 **Rarefaction curves of archaeal 16S rRNA and amoA gene sequences for maize and soybean rhizosphere samples.** Samples from ambient [CO_2_] plots are in grey and samples from elevated [CO_2_] plots are in black. The numbers at the ends of each curve identify the specific SoyFACE plot that each sample came from. 1∶1 lines, indicating infinite diversity, are also shown. OTUs were defined as groups of sequences sharing 97% 16S rRNA or *amo*A nucleotide sequence similarity.
(TIF)

Figure S2 **Rank‐relative abundance curves (on semi‐log axes) for OTUs of archaeal 16S rRNA and *amo*A gene sequences for maize and soybean rhizosphere samples.** Samples from ambient [CO_2_] plots are in grey and samples from elevated [CO_2_] plots are in black, as in Fig. S1.
(TIF)

Figure S3 **Phylogenetic trees of archaeal 16S rRNA gene sequences from rhizosphere soil samples from maize grown at ambient and elevated [CO_2_].** The colored bars represent the relative abundances of representative sequences from individual plots for each [CO_2_] treatment. Each colored bar in each phylogenetic tree represents data from a different plot. Starting from the root, the colors for the leaf ranges indicate crenarchaeota group 1.1a (green), crenarchaeota group 1.1c (dark blue), euryarchaeota (tan), and crenarchaeota group 1.1b (light blue). Clusters within crenarchaeota group 1.1b were further divided into arbitrarily named groups as shown in Fig. 2 (e.g. 1.1b_1). Only branches with bootstrap support >60 are shown, and identical branch lengths are shown for all branches and leaves.
(PDF)

Figure S4 **Percent of different archaeal lineages (identified in Figs. 2, S3) for which there was no statistical difference among plant/[CO_2_] combinations.** The abbreviations are as follows: maize ambient [CO_2_] (Ma), maize elevated [CO_2_] (Me), soybean ambient [CO_2_] (Sa), and soybean elevated [CO_2_] (Se). Mean values (+/− one standard deviation) are shown.
(TIF)

Figure S5 **Phylogenetic trees of *amo*A rRNA gene sequences from rhizosphere soil samples for each plant/[CO_2_] combination.** The colored bars represent the relative abundances of representative sequences from individual plots for each plant/[CO_2_] combination, as in Figs. 2, S3. Identical branch lengths are shown for all branches and leaves.
(PDF)

Table S1 **Number of reads obtained per sample for each gene and Chao1 estimates.**
(PDF)
