# Supplementary figures and images for: Response of Archaeal Communities in the Rhizosphere of Maize and Soybean to Elevated Atmospheric CO2 Concentrations
Source: PLoS One. 2010 Dec 29;5(12):e15897. doi: 10.1371/journal.pone.0015897 (PMC3012111; doi:10.1371/journal.pone.0015897)

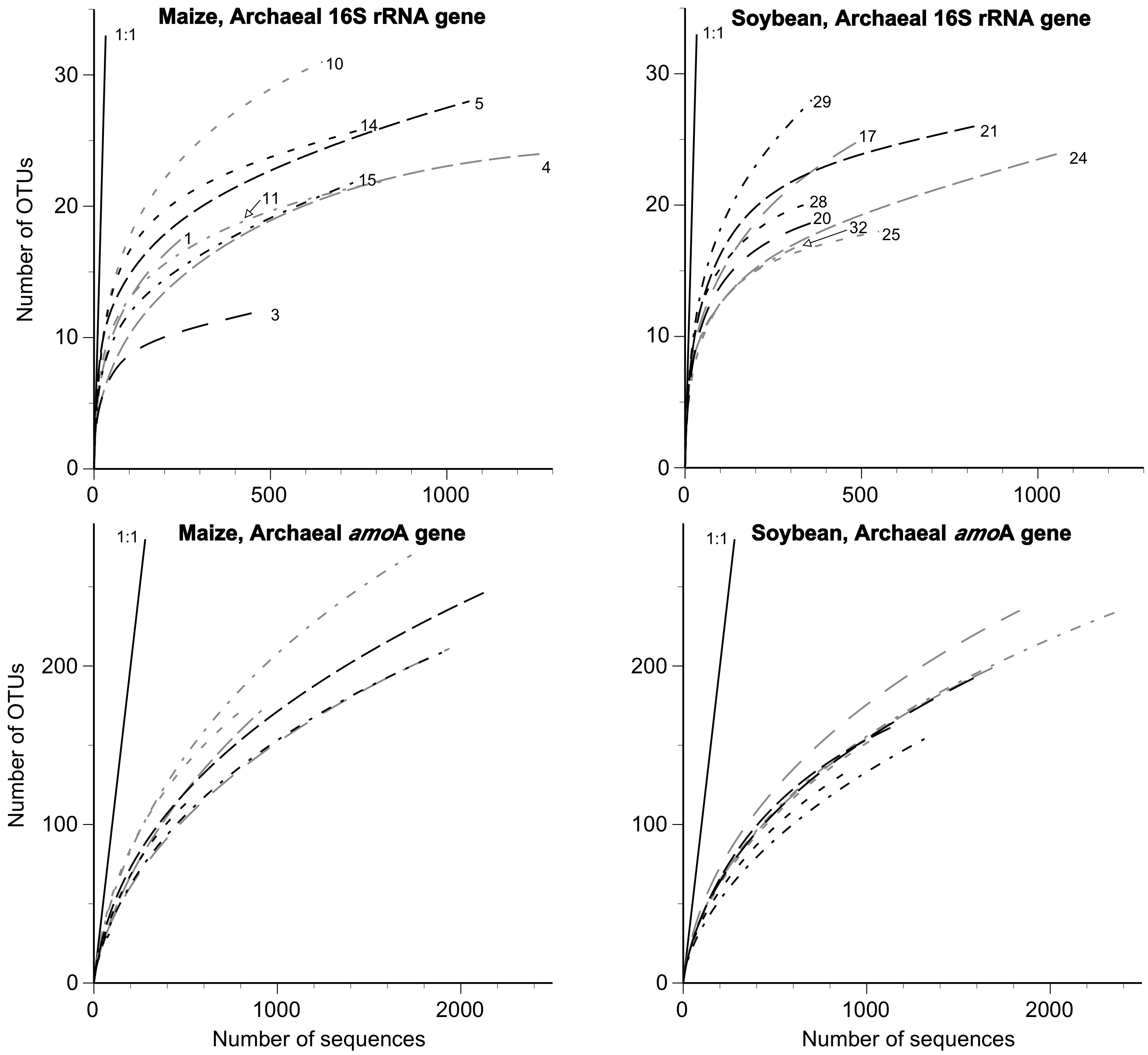

Supplement: Figure S1 — Rarefaction curves of archaeal 16S rRNA and amoA gene sequences for maize and soybean rhizosphere samples. Samples from ambient [CO2] plots are in grey and samples from elevated [CO2] plots are in black. The numbers at the ends of each curve identify the specific SoyFACE plot that each sample came from. 1∶1 lines, indicating infinite diversity, are also shown. OTUs were defined as groups of sequences sharing 97% 16S rRNA or amoA nucleotide sequence similarity. (TIF) [file pone.0015897.s001.tif]

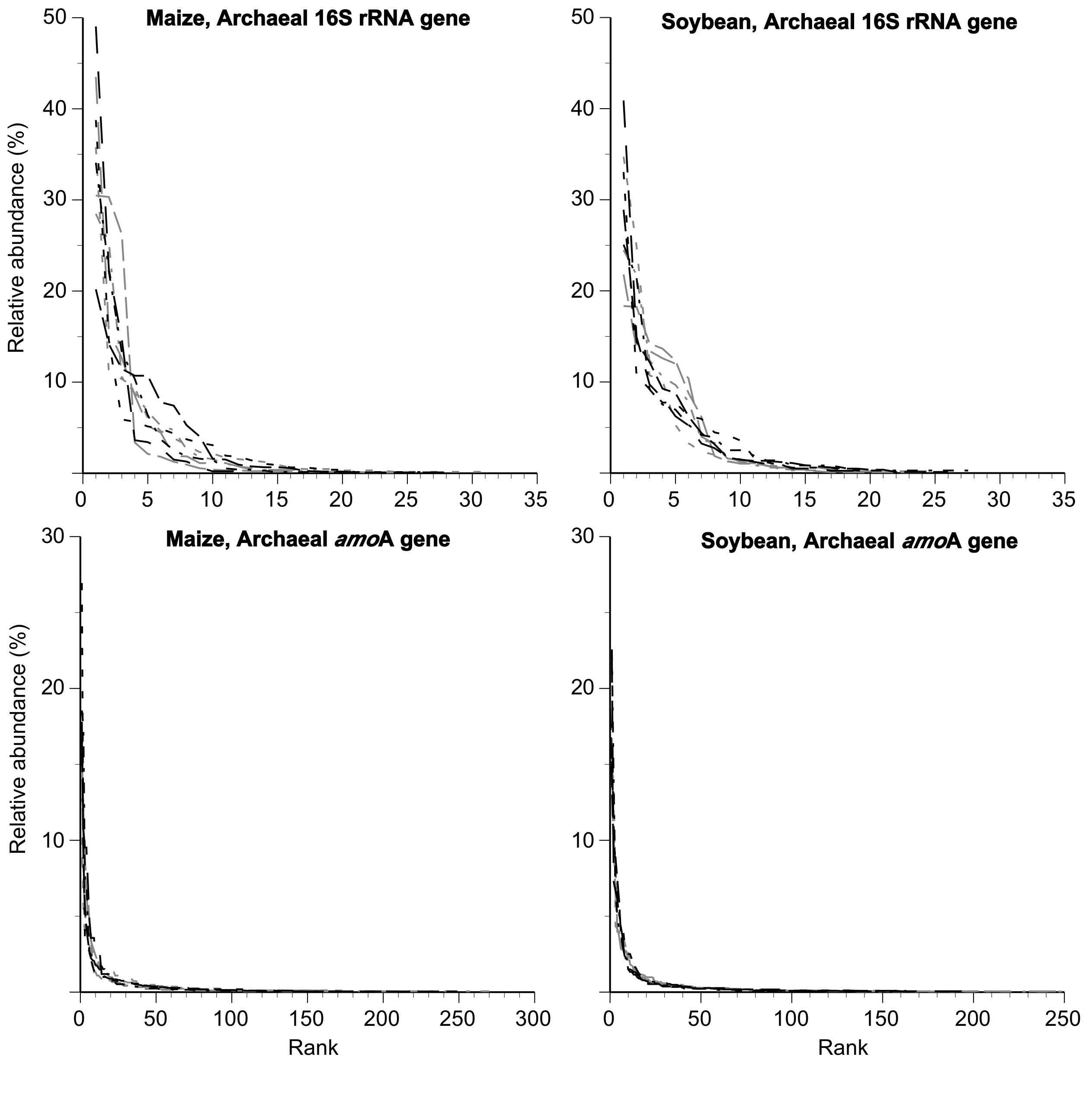

Supplement: Figure S2 — Rank‐relative abundance curves (on semi‐log axes) for OTUs of archaeal 16S rRNA and amoA gene sequences for maize and soybean rhizosphere samples. Samples from ambient [CO2] plots are in grey and samples from elevated [CO2] plots are in black, as in Fig. S1. (TIF) [file pone.0015897.s002.tif]

**A** Maize, ambient CO<sub>2</sub>

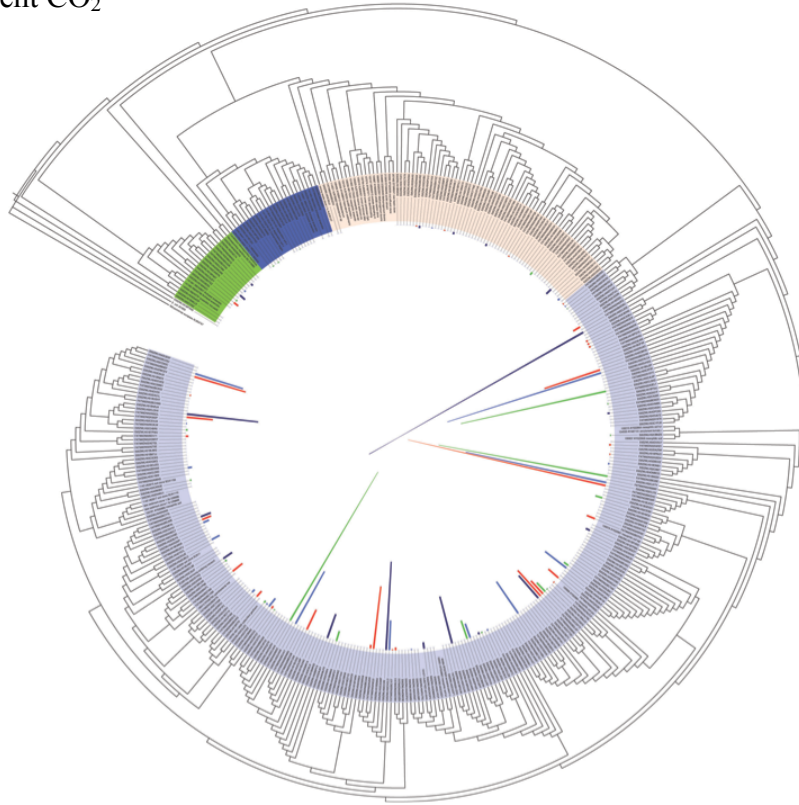

**B** Maize, elevated CO<sub>2</sub>

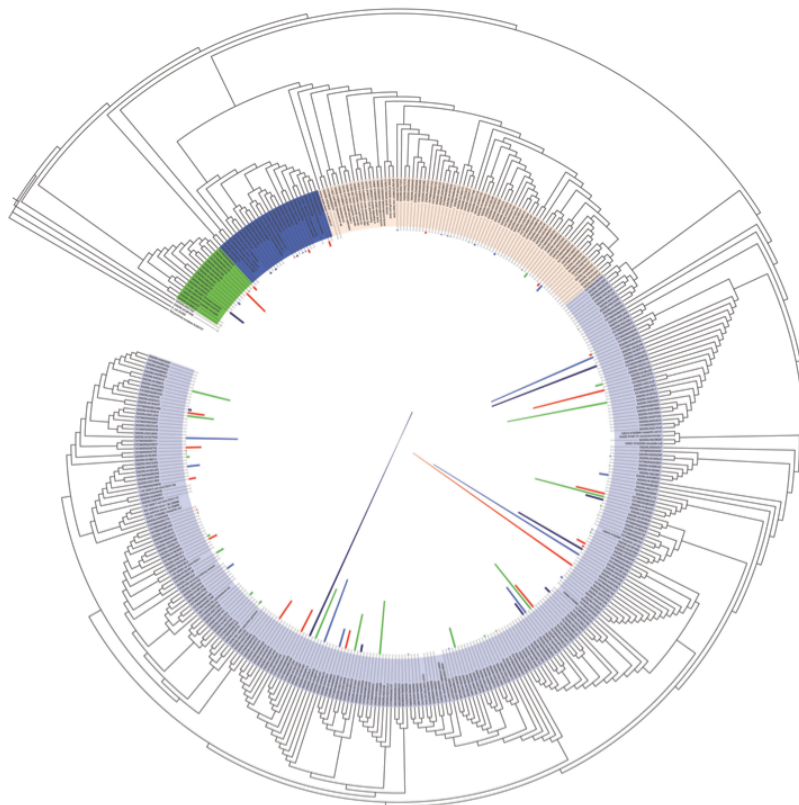

Supplement: Figure S3 — Phylogenetic trees of archaeal 16S rRNA gene sequences from rhizosphere soil samples from maize grown at ambient and elevated [CO2]. The colored bars represent the relative abundances of representative sequences from individual plots for each [CO2] treatment. Each colored bar in each phylogenetic tree represents data from a different plot. Starting from the root, the colors for the leaf ranges indicate crenarchaeota group 1.1a (green), crenarchaeota group 1.1c (dark blue), euryarchaeota (tan), and crenarchaeota group 1.1b (light blue). Clusters within crenarchaeota group 1.1b were further divided into arbitrarily named groups as shown in Fig. 2 (e.g. 1.1b_1). Only branches with bootstrap support >60 are shown, and identical branch lengths are shown for all branches and leaves. (PDF) [file pone.0015897.s003.pdf]

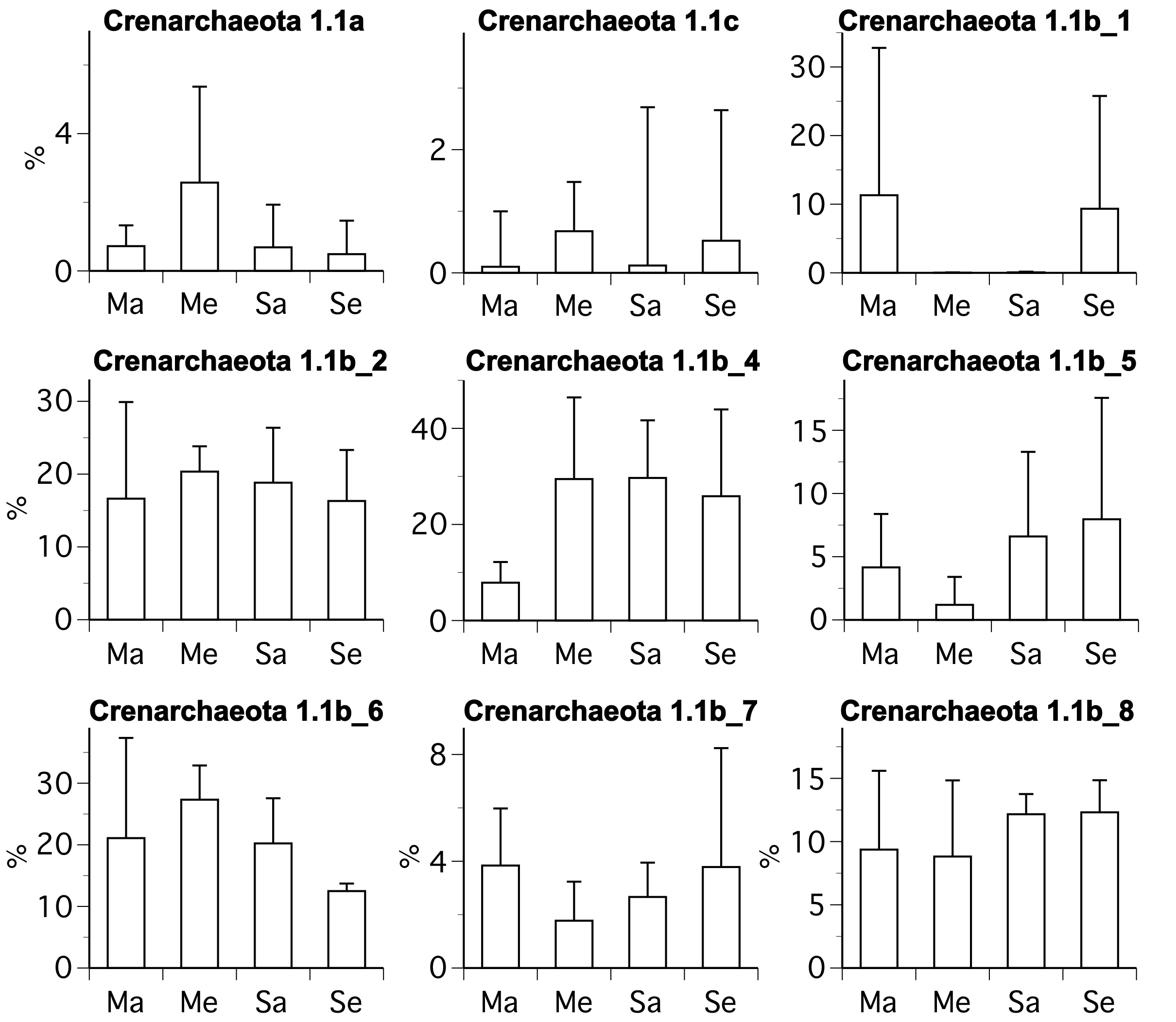

Supplement: Figure S4 — Percent of different archaeal lineages (identified in Figs. 2, S3) for which there was no statistical difference among plant/[CO2] combinations. The abbreviations are as follows: maize ambient [CO2] (Ma), maize elevated [CO2] (Me), soybean ambient [CO2] (Sa), and soybean elevated [CO2] (Se). Mean values (+/− one standard deviation) are shown. (TIF) [file pone.0015897.s004.tif]
