# Supplementary material for: Response of Archaeal Communities in the Rhizosphere of Maize and Soybean to Elevated Atmospheric CO2 Concentrations
Source: PLoS One. 2010 Dec 29;5(12):e15897. doi: 10.1371/journal.pone.0015897 (PMC3012111; doi:10.1371/journal.pone.0015897)

A Maize, ambient CO_2

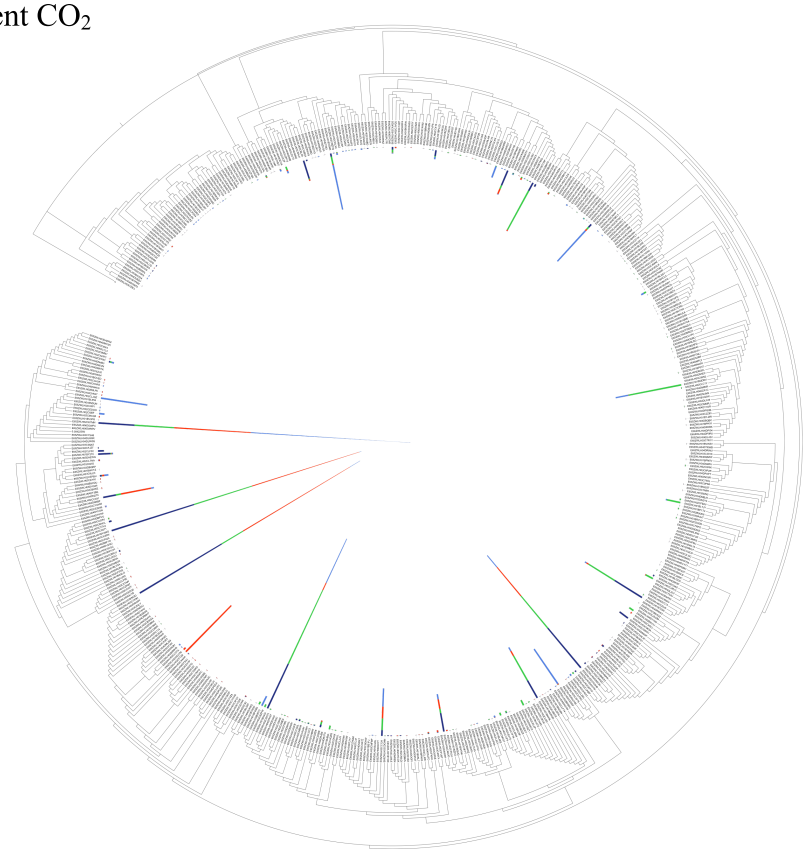

B Maize, elevated CO_2

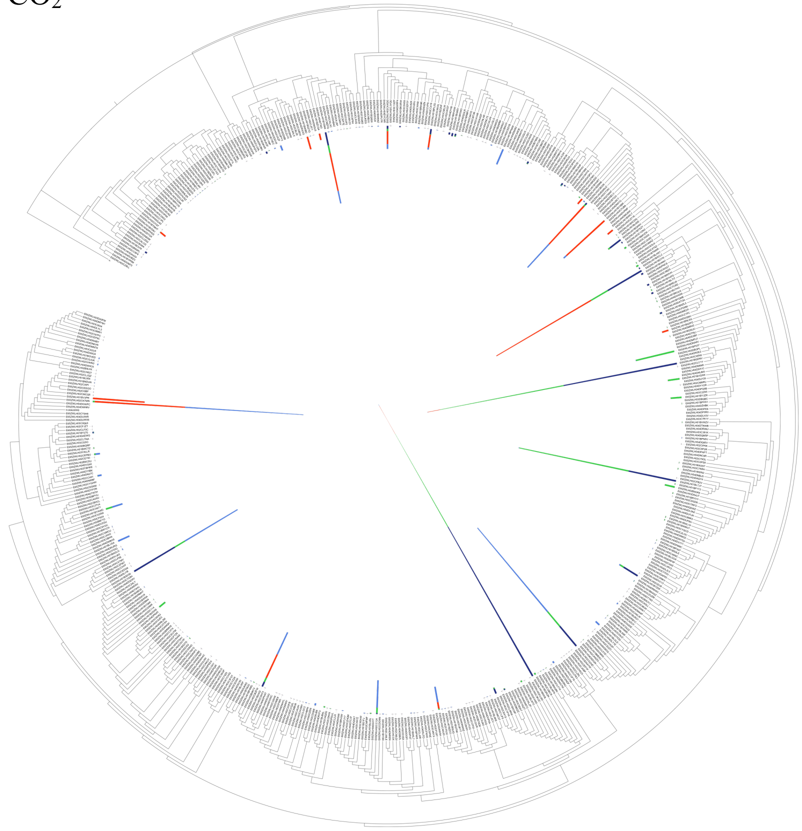

C Soybean, ambient CO_2

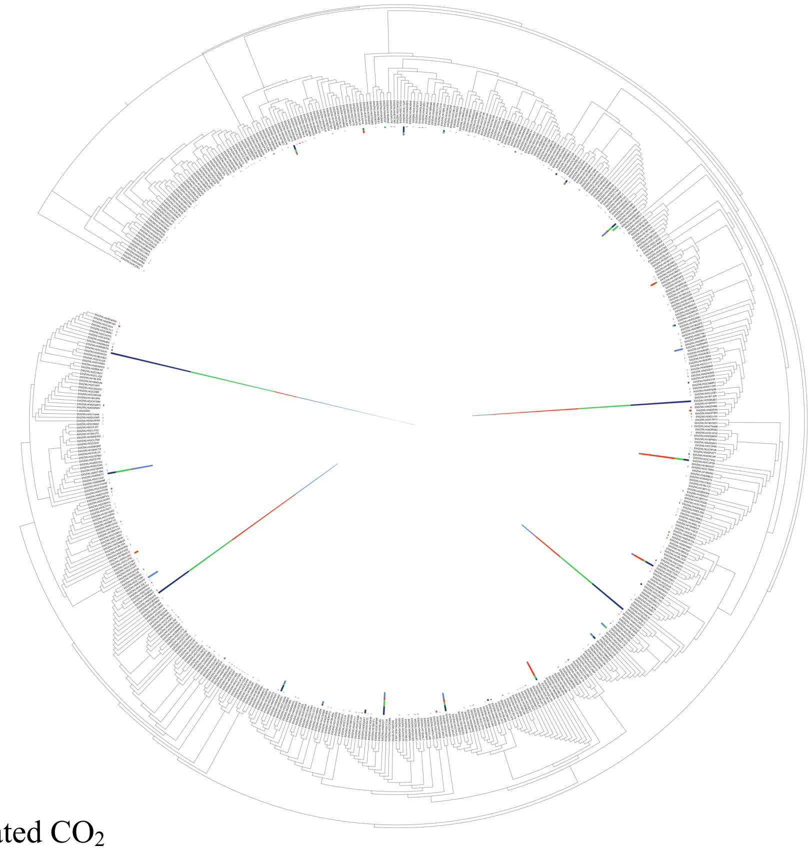

D Soybean, elevated CO_2

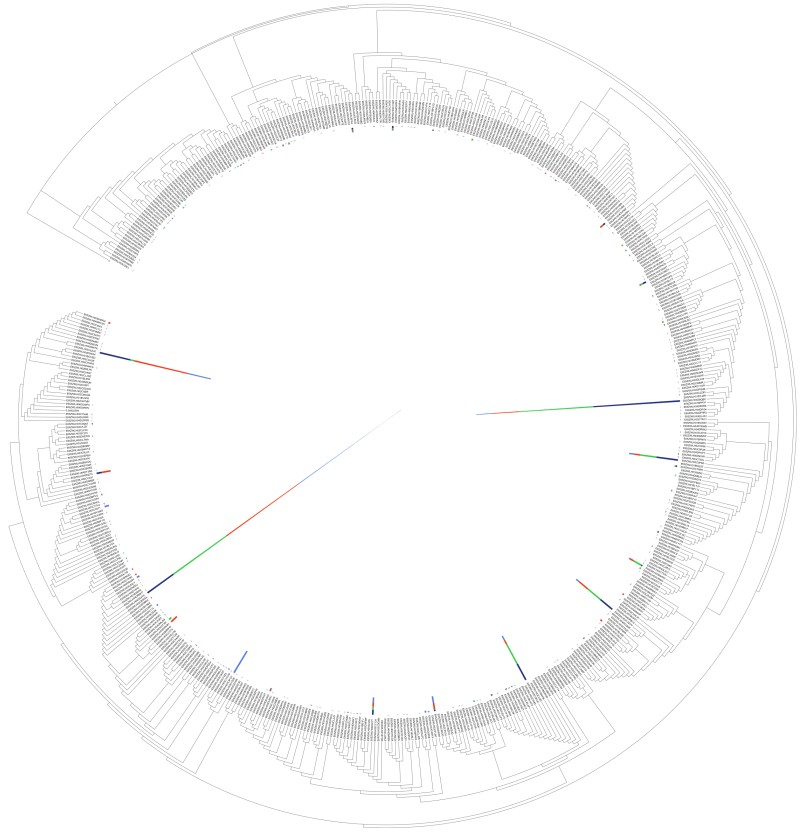

Supplement: Figure S5 — Phylogenetic trees of amoA rRNA gene sequences from rhizosphere soil samples for each plant/[CO2] combination. The colored bars represent the relative abundances of representative sequences from individual plots for each plant/[CO2] combination, as in Figs. 2, S3. Identical branch lengths are shown for all branches and leaves. (PDF) [file pone.0015897.s005.pdf]
