# Supplementary material for: Response of Archaeal Communities in the Rhizosphere of Maize and Soybean to Elevated Atmospheric CO2 Concentrations
Source: PLoS One. 2010 Dec 29;5(12):e15897. doi: 10.1371/journal.pone.0015897 (PMC3012111; doi:10.1371/journal.pone.0015897)
Supplement: Table S1 — Number of reads obtained per sample for each gene and Chao1 estimates. (PDF) [file pone.0015897.s006.pdf]

Supplemental Table 1.

		Archaeal 16S-V3		Archaeal *amo*A	
**Sample**	**[CO_2] treatment**	**# reads after QC[a]**	**Chao1 (95% confidence intervals)[b]**	**# reads after QC[a]**	**Chao1[c]**
Maize ring 1	ambient	270	26 (19-61)	1940	355
Maize ring 4	ambient	1274	24 (24-30)	915	292
Maize ring 10	ambient	649	37 (32-58)	825	270
Maize ring 11	ambient	845	23 (22-32)	1730	417
Maize ring 3	elevated	470	15 (12-37)	83	61
Maize ring 5	elevated	1066	35 (29-62)	2146	433
Maize ring 14	elevated	780	41 (29-95)	527	193
Maize ring 15	elevated	755	36 (25-89)	1908	319
Soybean ring 17	ambient	501	39 (28-92)	1834	392
Soybean ring 24	ambient	1071	38 (27-91)	1707	331
Soybean ring 25	ambient	551	20 (18-33)	1035	279
Soybean ring 32	ambient	334	19 (17-33)	2380	405
Soybean ring 20	elevated	402	20 (19-27)	1665	323
Soybean ring 21	elevated	821	36 (27-79)	1126	239
Soybean ring 28	elevated	337	21 (20-27)	875	235
Soybean ring 29	elevated	363	51 (33-125)	1347	368

[a]QC= quality-control; QC measures are described in the text.

[b]Determined using RDP's Chao1 estimator, as explained in the text.

[c]Determined using FastgroupII's Chao1 estimator, as explained in the text.
